# Technical note: A collision prediction tool using Blender

**DOI:** 10.1002/acm2.14165

**Published:** 2023-10-02

**Authors:** Gian Guyer, Silvan Mueller, Yanick Wyss, Jenny Bertholet, Remo Schmid, Marco F. M. Stampanoni, Peter Manser, Michael K. Fix

**Affiliations:** ^1^ Division of Medical Radiation Physics and Department of Radiation Oncology Inselspital Bern University Hospital, and University of Bern Switzerland; ^2^ Institute for Biomedical Engineering ETH Zurich and PSI Villigen Switzerland

**Keywords:** collision prediction, non‐coplanar radiotherapy, treatment planning

## Abstract

Non‐coplanar radiotherapy treatment techniques on C‐arm linear accelerators have the potential to reduce dose to organs‐at‐risk in comparison with coplanar treatment techniques. Accurately predicting possible collisions between gantry, table and patient during treatment planning is needed to ensure patient safety. We offer a freely available collision prediction tool using Blender, a free and open‐source 3D computer graphics software toolset. A geometric model of a C‐arm linear accelerator including a library of patient models is created inside Blender. Based on the model, collision predictions can be used both to calculate collision‐free zones and to check treatment plans for collisions. The tool is validated for two setups, once with and once without a full body phantom with the same table position. For this, each gantry‐table angle combination with a 2° resolution is manually checked for collision interlocks at a TrueBeam system and compared to simulated collision predictions. For the collision check of a treatment plan, the tool outputs the minimal distance between the gantry, table and patient model and a video of the movement of the gantry and table, which is demonstrated for one use case. A graphical user interface allows user‐friendly input of the table and patient specification for the collision prediction tool. The validation resulted in a true positive rate of 100%, which is the rate between the number of correctly predicted collision gantry‐table combinations and the number of all measured collision gantry‐table combinations, and a true negative rate of 89%, which is the ratio between the number of correctly predicted collision‐free combinations and the number of all measured collision‐free combinations. A collision prediction tool is successfully created and able to produce maps of collision‐free zones and to test treatment plans for collisions including visualisation of the gantry and table movement.

## 1 INTRODUCTION

Modern C‐arm linear accelerators (linacs) equipped with a multileaf collimator (MLC) support state‐of‐the‐art treatment techniques such as intensity modulated radiotherapy (IMRT) and volumetric modulated arc therapy (VMAT).^1^, ^2^ In recent years, non‐coplanar treatment techniques on C‐arm linacs enabled through table rotations were developed.^3^, ^4^ However, patient safety in terms of collision avoidance remains a concern. There are measures to prevent direct collisions, for example on a TrueBeam system (Varian Medical Systems, Palo Alto, USA) these include machine motion models, live view monitoring, touch guards and a laserguard.^5^ With these measures, the treatment delivery is ideally interrupted before a direct collision. However, potential subsequent replanning can cause delays in the treatment for several days in the worst case and a partially delivered plan is problematic for the therapeutic process. Thus, it is of great importance to include the information about collision‐free zones already in the treatment planning using a collision prediction tool to ensure deliverability of the treatment plan.

Several collision prediction tools for C‐arm linear accelerators have been developed in the past.^6^, ^7^, ^8^, ^9^, ^10^, ^11^, ^12^, ^13^, ^14^, ^15^, ^16^, ^17^ These collision prediction tools consist of a geometric model for the C‐arm linear accelerator and most of these tools also include a model for the patient.^7^, ^8^, ^9^, ^10^, ^11^, ^12^, ^13^, ^14^, ^15^, ^16^, ^17^ The geometric models of the C‐arm linac and table consist either of simple geometric shapes such as cuboids and cylinders,^6^, ^7^, ^9^, ^13^, ^15^ 3D meshes taken from measurements^11^, ^16^, ^17^ or detailed vendor‐provided machine data.^8^, ^10^, ^12^, ^14^ However, to our knowledge the laserguard is not incorporated in any of the published models.

In this work, we offer an implementation of a freely available collision prediction tool for a TrueBeam system using Blender. Blender is a free and open‐source computer graphics software,^18^ offering a modelling tool to create objects, a built‐in collision detection system between objects, a application programming interface (API) for easy automatization, and multiple render engines for visualisation of treatment plans. Nonetheless, Blender has to our knowledge never been used for a collision prediction tool in the field of radiotherapy.

## 2 METHODS

### 2.1 Blender model

A model of a TrueBeam system was created in Blender. For this, measurements on a machine were taken with a tape measure and the machine was recreated using the integrated modelling tools inside Blender. A model for the gantry, the collimator, the table base and the table top was created. Additionally, the sensitive area of the laserguard of the TrueBeam was modelled using information from the TrueBeam manual as well as from measurements. The laserguard detects collisions using an infrared laser scanning device on a plane between the collimator and the patient. The sensitive area of the laserguard is V‐shaped with a notch in the middle.^5^ Because collision interlocks between the gantry and the table stop the treatment a few centimeters before the actual collision, additional enveloping structures around the table top with 2 cm extra and around the table base with 5 cm extra were created. The extra distances were determined by measuring the smallest distance between the gantry and the table after triggering a collision interlock. Additionally, a carbon fiber head plate (Civco Medical Solutions, Kalona, USA) used for fixation of thermoplastic head masks was modelled.

For the patient model, a patient library was generated based on average human proportions. For this, the proportions of a human base‐mesh was transformed using MakeHuman, a free and open‐source 3D character modelling software.^19^ The proportions were fitted to the 25th and 75th percentile of measurements of US adult males and females taken from Segars et al.^20^ The 25th percentile is denoted the small size and the 75th percentile is denoted the large size for male and female patients, respectively. The patient library consists in total of 8 patient models, with two positions for each size and sex of the patient model: head‐first supine with the arms down and head‐first supine with the arms up above the head.

### 2.2 Collision‐free zones

To calculate the collision‐free zones, a python script using the Blender API was written, which simulates all possible gantry‐table angle combinations according to a specified resolution based on user input data. Using bounding volume hierarchies (BVHs), the script determines overlap of the 3D meshes of gantry and laserguard with the 3D meshes of the enveloping structures for table and patient model. Optionally, the user inputs safety margins in which case a collision is considered if the minimum distance between the 3D meshes is smaller than this margin. The initial table positions are specified with one of two ways:

1. The table positions are specified relative to a reference point on the head plate by measuring the difference between the treatment plan isocenter and the reference point in *x*, *y* and *z* coordinates in the patient coordinate system. This difference is added to the table positions when the reference point is in the isocenter.
2. The table positions are specified in absolute table values.

Because the table position varies between fractions, tolerances for pitch, roll and rotation and lateral, vertical and longitudinal table axes can be specified. The table position is shifted or rotated from its initial position plus and minus the specified tolerance in each axis. The tool then checks for collision also for each combination of tolerance shifts and rotations.

A map with the predicted collision‐free zones is created as output. To ease the specification of the input data, a graphical user interface (GUI) is created using the scripting API of a research version of the Eclipse treatment planning system (Varian Medical Systems, Palo Alto, USA).

### 2.3 Validation

To validate the geometric model, collision predictions for each gantry‐table angle combination with a 2° resolution were generated for one initial table position with two different setups:

1. Laserguard enabled, no patient model, head plate disabled, no additional margin, zero tolerance.
2. Laserguard enabled, small male patient model with arms down, head plate enabled, no additional margin, zero tolerance.

On a TrueBeam, the table was moved to the initial table position and the table and gantry were rotated to each gantry‐table angle combination with a 2° resolution once with the table alone and once with an Alderson Radiation Phantom (ART) positioned on the table. Each of the gantry‐table angle combination was manually checked for a collision interlock either between the laserguard and the table or the gantry and the table.

The predicted and measured collision interlocks were evaluated for each gantry‐table combination using the evaluation metrics shown in Table 1.

**TABLE 1:** The evaluation metrics between the predicted and measured collision interlocks.

	Collision predicted	
Collision measured	Yes	No
Yes	True positive (TP)	False negative (FN)
No	False positive (FP)	True negative (TN)

The number of TP, FN, FP and TN values over all gantry‐table combinations were determined and the true positive rate (TPR) and true negative rate (TNR) were calculated using the following equations.

(1) $$TPR=\frac{\#TP}{\#TP+\#FN}*100\%$$

(2) $$TNR=\frac{\#TN}{\#TN+\#FP}*100\%$$

### 2.4 Collision check of treatment plans

As a second application of the collision prediction tool, the fields of a treatment plan can be checked for collisions. The fields are checked for collision by giving the static or dynamic table angle and position, gantry angle and collimator angle as additional input. The tool then checks for collisions for the static position or along the dynamic path of the field using the same method as described above.

For demonstration, a dynamic trajectory radiotherapy (DTRT) path with dynamic gantry rotation, table rotation and collimator rotation was created retrospectively for a head and neck (H&N) case using the method described by Fix et al.^21^ Additionally, a second path was created with an increased source‐to‐target distance (STD) of 110 cm by translating the table 10 cm away from the gantry in the beam direction along the whole path as described by Guyer et al.^22^ The initial table position was set using the relative method with the reference point on the head plate. The large male patient model with the arms down was chosen and an additional margin of 3 cm was set for the patient model. For pitch, roll and rotation axes, a tolerance of 2° was used and for lateral, longitudinal and vertical axes a tolerance of 3 mm was used. The tolerances were determined by retrospectively reviewing the clinically applied treatment plan and taking the largest applied setup table shifts for each axis over all fractions.

## 3 RESULTS

### 3.1 Blender model and GUI

**FIGURE 1:**
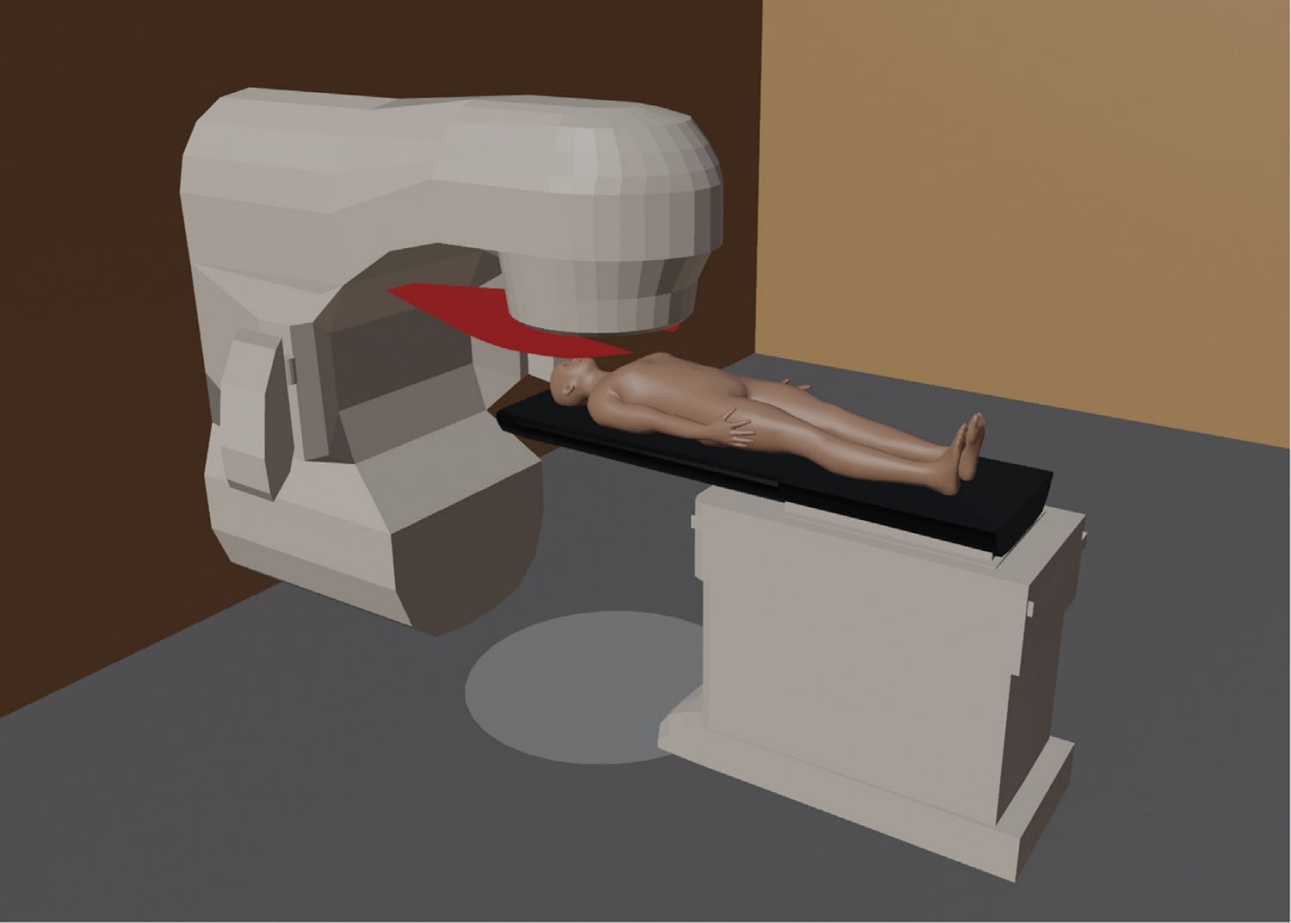
Screenshot of the Blender model including gantry and table stand in light grey, table top in black, laserguard in red and patient in skin color.

**FIGURE 2:**
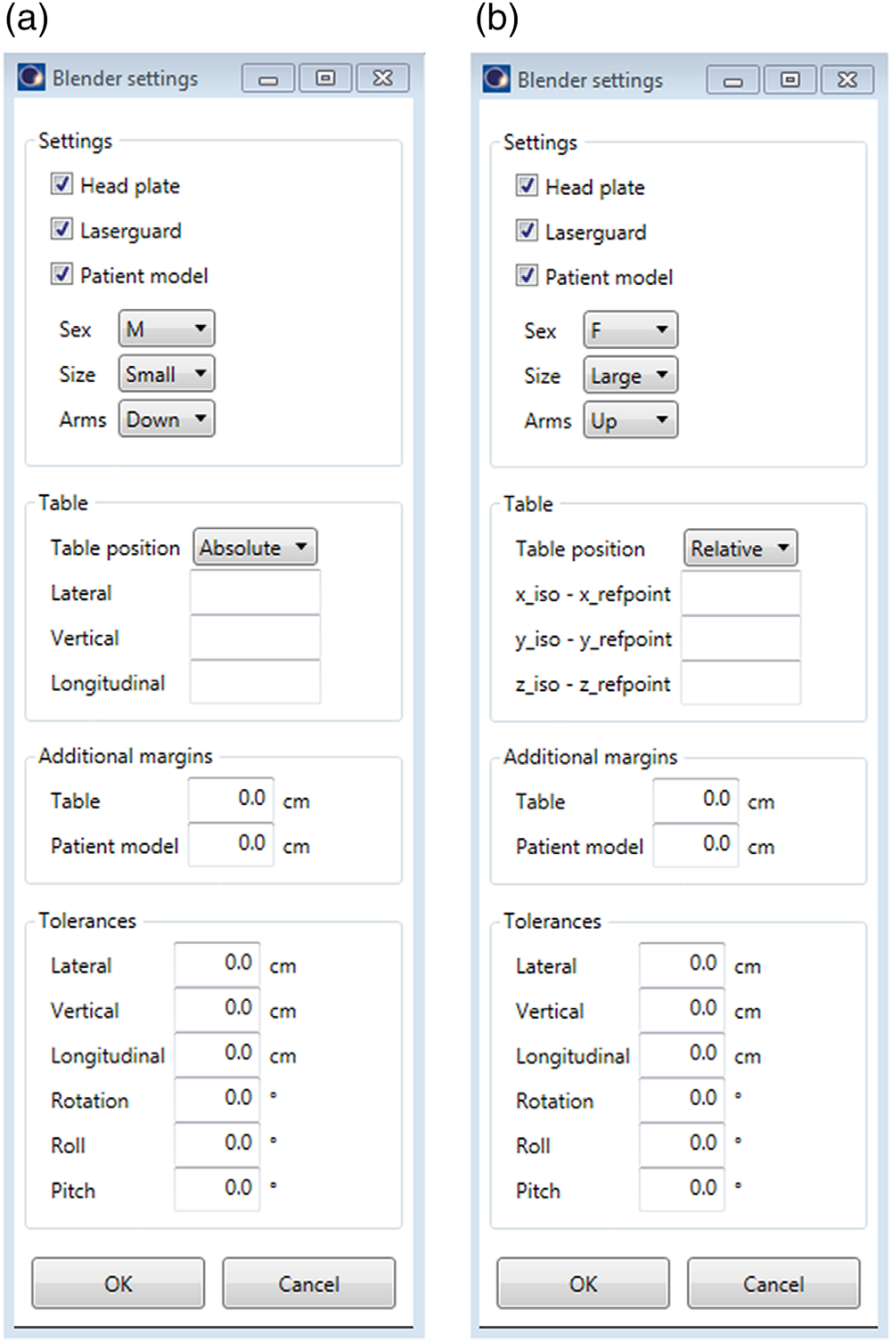
Two screenshots of the GUI to specify input for the collision prediction tool. The selected option for specifying the table position is absolute in (a) and relative in (b). GUI, graphical user interface.

A screenshot of the Blender model is shown in figure 1 and two screenshots of the developed GUI using Eclipse scripting API are shown in Figure 2. The GUI allows the user to disable or enable the head plate and laserguard, to choose a patient model, to set the table positions in absolute values or relative to a head plate, to set additional safety margins and to set tolerances for the table position and rotation. The model and source code are freely available at https://github.com/gianguyer/collisionCheck.

### 3.2 Validation

**FIGURE 3:**
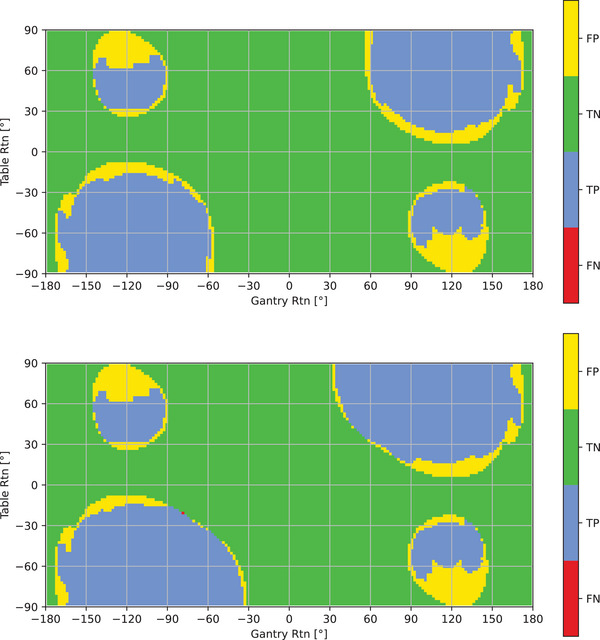
Map of gantry‐table combinations resulting in FP, TN, TP and FN values when comparing predicted and measured results for the setup without any phantom on the table (a) and with the ART phantom on the table (b). ART, Alderson radiation phantom; FN, false negative; FP, False positive; TN, true negative; TP, true positive.

In Figure 3, a gantry‐table map is shown with an evaluation between the measured and predicted collisions for all gantry‐table angle combinations. For the setup without phantom, the TPR is 100.0% and the TNR is 88.8%. For the setup with the ART phantom, the TPR is 99.9% and the TNR is 89.1%.

### 3.3 Collision check of plans

**FIGURE 4:**
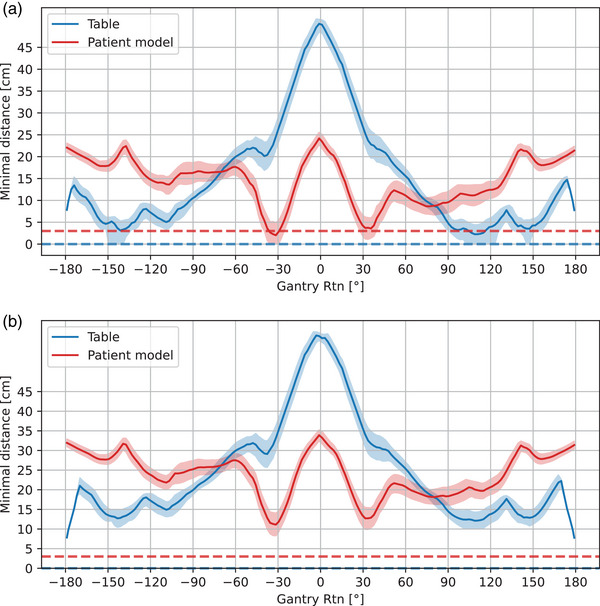
Minimum predicted distances between the gantry and the table and the patient model for a DTRT path for a H&N case with an STD of 100 cm (a) and an STD of 110 cm (b). The minimum distances over all combinations of axes tolerances are shown in bands. The nominal combination, i.e. the situation with tolerance values of zero, is shown as a solid line. In blue and red dashed horizontal lines, the additional margin between gantry and table and gantry and patient model is indicated, respectively. DTRT,dynamic trajectory radiotherapy; STD, source‐to‐target distance.

A DTRT path with a STD of 100 cm and a DTRT path with an extended STD of 110 cm were checked for collisions. The minimum distances between the gantry and the table and the gantry and the patient model are shown in Figures 4a and b for STD of 100 and 110 cm, respectively. As can be seen, the tool predicts collisions with the table for the path with an STD of 100 cm for certain combinations of axes tolerances. For the path with STD of 110 cm, the distance between the gantry and table and gantry and patient model is above the specified margins for the whole path and all combinations of tolerances. A video of the gantry and table movement simulation inside Blender is provided in the supplementary material.

## 4 DISCUSSION

Using Blender, a model of a C‐arm linac was successfully created. Blender is a free and open‐source software, which allows broad access to a powerful computer graphics software toolset. The python API allows for easy automatization of simulations inside Blender. Using the python API, two applications for collision prediction were created. First, calculation of collision‐free zones, which allows to avoid unfeasible gantry‐table combinations during the treatment plan creation. Second, a plan check feature, which allows for checking a path of a treatment plan for specific table positions. This second feature is especially helpful, if the table position deviates from the expected position at the planning stage or if the plan was not created using the collision‐free maps. Additionally, tolerances for the table position can be considered in both applications. The tolerances allow to consider the daily table shifts in the collision prediction. In this work, the laserguard is integrated into the collision prediction tool using a model of the sensitive area of the laserguard, which is an advantage over collision prediction tools which use vendor provided data without any laserguard^10^, ^12^, ^14^ or which use a model based on 3D surface scans.^11^

In the collision check of plans feature, also videos of the simulation of the gantry and table movement are created, similar to the work of Suriyakumar et al and Wang et al.^15^, ^17^ This has the benefit, that the dynamic rotations and translations of gantry, collimator and table in DTRT plans are visualisable prior to the delivery. Additionally, precarious areas where the table or patient may come close to the gantry are easier identifiable. Furthermore, the simulations can serve as a visual aid in explaining the radiotherapy treatment to patient and staff, also to reduce potential patient anxiety.^23^, ^24^, ^25^

The Blender model was validated for one lateral, vertical and longitudinal table position by comparing predictions of collisions for all gantry‐table angle combinations to measurements. For a collision prediction tool, it is important, that the TPR is practically 100%, because otherwise collision might still occur which the tool did not predict. On the other hand, a low TNR indicates that the collision‐free space is reduced unnecessarily. In the balance, safety (a high TPR) is more preferred. In the validation, some false negatives did occur for collision interlocks with the laserguard but overall, the model is deemed adequate. The TNR is lower than the TPR due to some false positive areas around the smaller collision zones. This is mainly due to the complex shape of the collimator and the applicator mounting and the fact that the sensitive area of the laserguard has a notch, which is difficult to model correctly.

In this work, the patient is modelled by relying on averaged data of human proportions and predefined patient positionings. Because of this simplification, collision interlocks between the patient and gantry might still occur with the custom positioning of the patient, such as having their arms in a different location, and not a wide enough margin around the patient model is applied. To model the patient more accurately, others have suggested to use a commercial 3D surface scanner.^8^, ^11^, ^13^, ^14^ With this, the patient's anatomy as well as the custom positioning of each patient can be considered. Furthermore, the patient's surface can be scanned daily and a collision prediction with the daily scan can be performed. Because Blender can import STL files, which is a standard output format of 3D surface scanners, an extension of the Blender collision prediction tool for automatic import and registration of patient surface scans is feasible in the future.

## 5 CONCLUSION

In this work, a collision prediction tool using Blender is successfully developed. The prediction tool is able to predict collision zones as well as check treatment plans for collisions including laserguard and positioning variations. Additionally, videos of the gantry and table movement of treatment plans can be produced and minimum distances between gantry, table and patient model are visualised. The tool facilitates a smoother clinical workflow with less replannings due to collision interlocks.

## AUTHOR CONTRIBUTIONS

Michael K. Fix and Peter Manser concieved the initial idea. Gian Guyer, Silvan Mueller, Remo Schmid and Yanick Wyss performed the measurements for this study. Gian Guyer and Yanick Wyss created the tool and wrote the code. Gian Guyer, Silvan Mueller and Jenny Bertholet performed the validation. Marco F. M. Stampanoni, Peter Manser and Michael K. Fix supervised the project. Gian Guyer wrote the manuscript with critical feedback from all co‐authors.

## CONFLICT OF INTEREST STATEMENT

The authors declare no conflict of interest.

## Supporting information

Supplementary material
